# Perception of Human Papillomavirus Infection, Cervical Cancer and HPV Vaccination in North Indian Population

**DOI:** 10.1371/journal.pone.0112861

**Published:** 2014-11-11

**Authors:** Showket Hussain, Vilas Nasare, Malasha Kumari, Shashi Sharma, Mohammad Aijaz Khan, Bhudev C. Das, Mausumi Bharadwaj

**Affiliations:** 1 Division of Molecular Genetics & Biochemistry, Institute of Cytology & Preventive Oncology (ICMR), Noida, Uttar Pradesh, India; 2 Division of Clinical Oncology, Institute of Cytology & Preventive Oncology (ICMR), Noida, Uttar Pradesh, India; 3 Division of Biostatistics & Epidemiology, Institute of Cytology & Preventive Oncology (ICMR), Noida, Uttar Pradesh, India; 4 Division of Behaviour Oncology, Institute of Cytology & Preventive Oncology (ICMR), Noida, Uttar Pradesh, India; 5 Division of Molecular Oncology, Institute of Cytology & Preventive Oncology (ICMR), Noida, Uttar Pradesh, India; Istituto Nazionale Tumori, Italy

## Abstract

**Background:**

Human Papillomavirus (HPV) -associated cervical cancer is the second-most common cancer in women worldwide but it is the most frequent gynaecological cancer and cancer associated death in India women. The objective of this study was to assess knowledge about cervical cancer, HPV, HPV vaccine, HPV vaccine acceptance among school and undergraduates students and their parent’s perception about acceptance of HPV vaccine in Northern part of India (Delhi and NCR regions).

**Materials and Methods:**

A qualitative questionnaire based survey among 2500 urban/rural students aged 12–22 years was conducted.

**Results:**

Overall, a low frequency (15%) of HPV and cervical cancer awareness was observed in students and their parents. However, the awareness was much higher in females belonging to urban setup compared to boys with a perception that HPV causes cervical cancer in women only. Additionally, only (13%) participants who were aware of cervical cancer and HPV) were willing to accept HPV vaccination. Apparently, parents of female students were two times more willing to accept HPV vaccination for their ward than male students (p<0.001; OR 95%CI = 2.09 (1.58–2.76).

**Conclusion:**

Cervical cancer and HPV awareness among school, undergraduate students and also to their parents was found to be very low in this part of India. The level of awareness and education appears to be insignificant determinants in rural compared to urban setup. Better health education will be needed to maximize public awareness for cervical cancer prevention.

## Introduction

Cancer of the uterine cervix is the second-most common cancer in women worldwide but it is the most common health hazard in India [1]. 80% of sexually active women infected with persistent HPV infection leads invasive cervical cancer [1], [2], [3]. However, reduction of morbidity/mortality due to cervical cancer is early detection and treatment of cervical precancerous lesions. In developed countries, the mortality has reduced due to introduction of population-based cytological screening programme using Pap test [4]. In addition, screening for cervical infection of HPV has proved to be more effective and reliable [5].

Recently, two HPV vaccines quadrivalent “Gardasil” and bivalent “Cervarix” have been shown to be highly effective in preventing infection with high-risk type HPV16 and HPV18, the two most common oncogenic types. As these vaccines are highly effective before exposure to HPV, current guidelines prioritise adolescent girls as primary target group for HPV vaccination [6].

Though adolescents are the prime candidates for prophylactic HPV vaccination, several studies have shown that adolescents had very little knowledge about HPV vaccination [7], [8], [9]. The deficiency of such knowledge might adversely affect the vaccine acceptance. Therefore, there has been an exponential increase in the number of studies exploring acceptability of the vaccine since it became commercially available. Many studies have focused on parental willingness to vaccinate their children [1], [10], [11], [12], [13], [14]. But only few studies have evaluated the knowledge of HPV vaccine, attitudes and intentions of young women [15], [16], [17], [18]. Recent reviews [19], [20], [21], [22] showed that acceptance of the vaccine is high among those who are aware of the relationship between HPV and cervical cancer.

The deficiency of knowledge of causal relationship between HPV and cervical cancer might adversely affect the vaccine acceptance in India. Several studies have been undertaken to evaluate women’s awareness and knowledge level about cervical cancer and HPV vaccine [23], [24], [25]. A report from rural India among nursing staff showed that 74% know that pap smear is used for detection of cervical cancer but only 59% know that pap smear can detect both cancerous as well as precancerous lesions of cervix. Surprisingly, only 18% know about HPV vaccine [25].

Preliminary study among 8–17 years old adolescents and their parents in India, suggested that majority of them are unaware of cervical cancer, HPV and HPV vaccines [26]. In view of this, we conducted a study among students from high-school up to undergraduate students to evaluate their knowledge about knowledge of cervical cancer, HPV awareness, willingness of HPV vaccine acceptance among adolescents and their parents and to identify predictors of girls receiving the HPV vaccine.

## Materials and Methods

A pre-designed questionnaire-based survey was conducted and a total of 2600 participants aged between 12–22 years were enumerated from June 2009–June 2011 that included both urban and rural school population of Noida and Delhi. Out of 2600 participants, only 2500 participants completed the questionnaire. The study was approved by Ethics Committee of Institute of Cytology & Preventive Oncology (ICPO/IEC/2008-09/05). Written consent was taken from all participants and guardians in case of minors.

First part of the questionnaire was to collect information on age, sex, study stream, level of study, place of permanent residence, family income, family size and religion etc. were noted. The remaining part a self-administered questionnaire was used for this study, which were to be filled in individually by the participants under the strict vigilance of the teachers/researchers/mentors which serves as invigilators to monitor the influence on answers by the students. The questionnaire contained questions regarding knowledge of cervical cancer, HPV and awareness about HPV vaccine. In addition, there is question on parent’s opinion about HPV vaccine acceptance. The questions were developed based on previous established facts for cervical cancer [27], [28], [29], [30]. The most of the questions “yes”, “no”, or appropriate multiple choices were given as answers. The data from questionnaire were processed anonymously. Furthermore, the data was again validated by revisiting the same school after one, three and six months of the 1^st^ visit to access the knowledge and awareness among participants. Data were analysed using SPSS software version 14.0 to assess the association between demographic factors and HPV awareness and cervical cancer.

## Results

A total of 2600 students participated in the study. Sixty eight (68) participants did not complete the personal information details and thirty two (32) were having incomplete questionnaires. Therefore, a total of 2500 participants could complete the questionnaire, out of which 930 (37.2%) were male and 1570 (62.8%) were female. Most of them 56% were from rural set up where as 44% were of urban region. Most of students 58.7% were in the age group of 19–22 years, 28.5% belonged to16–18 years, while 12.8% were of age group 12–15 years. The participation rate of female students was higher (62.8%) as compared to male students (37.2%). Two hundred eighty six (11.44%) students were in the education group of 6–8^th^ standard, eight hundred thirteen (32.52%) students were of 10–11^th^ standard whereas fourteen hundred and one (56.04%) students were of 12^th^ standard as well as of undergraduate level respectively. Further stratification of data with respect to religion revealed a higher participation rate of Hindu students (68.56%) which was evident as majority of Hindu population is dominant in these areas whereas 31.44% were of Muslim students. Detailed demographic characteristics of the participants are presented in Table 1.

**Table 1:** Demographic characteristics of the population studied.

Characteristics	No. ofParticipants (N = 2500)	PercentDistribution (%)
**Age**		
12–15	320	12.8
16–18	713	28.52
19–22	1467	58.68
**Sex**		
Female	1570	62.8
Male	930	37.2
**Education**		
Standard 6–8	286	11.44
Standard 9–11	813	32.52
Standard 12-undergraduate	1401	56.04
**Religion**		
Muslim	786	31.44
Hindu	1714	68.56

Table 2, 3 and 4 describes the awareness of the participants.

**Table 2:** Awareness about cervical cancer, HPV and HPV vaccine.

Parameters	Awareness among participants	
	Frequency	Percentage
Knowledge about cervical cancer (n = 2500)	375	15
Knowledge about HPV infectioncauses genital cancer (n = 375)	275*	73.3
HPV vaccine awareness (n = 275)	225	81.8
HPV vaccine willingness (n = 275)	175**	63.6
Parents opinion about HPV vaccine (n = 2500)	325***	13

*Number indicate participants who had knowledge about cervical cancer and know the causative agent.

**Number indicate participants who had both knowledge and know the causative agent.

***General opinion among all participants.

**Table 3:** Comparison of awareness among Females and Males.

Parameters	Sex		P-value	Odd ratio (95%CI)
	Female	Male		
**Knowledge about cervical cancer (n = 375)**	257(68.5%)	118(31.5%)	0.13	1.35 (1.06–1.72)
**Knowledge about HPV infection causes genital cancer (n = 275)**	172(62.5%)	103(37.4%)	0.93	0.99 (0.76–1.29)
**HPV vaccine awareness (n = 225)**	162(72%)	63(28%)	**0.004**	1.57 (1.14–2.14)
**HPV vaccine willingness (n = 175)**	123(70.2%)	52(29.7%)	**<0.001**	5.2 (3.45–9.01)
**Parents opinion about HPV vaccine (n = 325)**	248(15.79%)	77(8.27%)	**<0.001**	2.09 (1.58–2.76)

**Table 4:** Awareness about cervical cancer and HPV according to living area.

Factors	Knowledge	Living area		P-value	Odd ratio; (95%CI)
		Urban	Rural		
**Knowledge about** **cervical cancer (n = 375)**	Yes	235 (62.6)	140 (37.3)	**<0.001**	2.82 (2.07–3.83)
**Knowledge about HPV** **infection causes genital cancer (n = 275)**	Yes	172 (62.5%)	103 (37.45%)	0.02	1.36 (1.04–1.77)
**HPV vaccine awareness** **(n = 225)**	Yes	143 (63.5%)	82 (36.45%)	0.02	1.41 (1.05–1.89)
**HPV vaccine willingness** **(n = 175)**	Yes	113 (64.5%)	62 (35.4%)	0.02	1.47 (1.05–2.05)
**Parents opinion about HPV** **vaccine (n = 325)**	Yes	213 (65.53%)	112 (34.46%)	**<0.001**	1.58 (1.23–2.04)

### Knowledge about cervical cancer

In order to examine the knowledge/awareness about cervical cancer, HPV and HPV vaccines, a short type questions in local as well as in English language were asked to the students and their parents (Table 2). In general, three hundred seventy five (15%) participants were only well aware of cervical cancer. Of these, two hundred fifty seven (69%) female students were aware of cervical cancer whereas 118 (31%) male students knew about cervical cancer. A significant correlation of cervical cancer awareness was observed with respect to female students (p<0.05) and this could be attributed due to more female participation. Thus, cervical cancer knowledge was apparently more in female participants when compared to male participants (p<0.05, OR = 1.35, 95%CI = 1.06–1.72) (Table 3). Furthermore, data demonstrate that 235 students (63%) belonged to rural area whereas 140 (37%) were of urban and had a knowledge about cervical cancer. On the other hand, a significant difference was also observed when the comparison was made between rural and urban residence of students. Certainly, a more knowledge about cervical cancer was observed in participants who belonged to urban setup, (p<0.001, OR = 1.38; 95%CI = 1.10–1.75) (Table 4).

### Knowledge of HPV causes genital cancer

Of the total participants (375) who had knowledge about cervical cancer, two hundred seventy five (73%) participants were having knowledge that cervical cancer is caused due to HPV infection (Table 2). A significant difference was observed between male: female and rural vs urban participants, as one hundred seventy two (63%) females and one hundred three (37%) males were aware that HPV infection causes genital cancer (Table 3).

### HPV vaccine awareness

Furthermore, the students were asked to know whether or not they are aware that HPV vaccines are commercially available, two hundred twenty five participants were aware of availability of vaccines. Among them, one hundred sixty two (72%) were female students and sixty three (28%) were male students. Therefore, the correlation of gender of the students with HPV vaccine awareness was observed and found to be statistically significant (p = 0.004; OR = 1.57, 95%CI = 1.14–2.14). Interestingly, vaccine availability awareness was also attributed to urban population 65% (143/225) (Table 4).

### HPV vaccine willingness

Students were educated about the risk factors associated with cervical cancer and how it could be prevented through vaccination and other practices, the willingness of HPV vaccination was found to be 70% in females and 64% in urban populations (Table 3 and Table 4).

### Parent’s opinion about HPV vaccine

Last but not the least, parents opinion was also taken into consideration with respect to vaccine implementation program in North-Indian population. 13% of the parents agreed to vaccinate their wards (Table 2).

### Validation of questionnaire

After completion of questionnaire by participants, a brief power point presentation was addressed among all subjects which included introduction to cervical cancer, incidence rate both in India and world wide, diagnostic modalities: Pap-test, liquid based cytology (LBC), visual inspection (VIA, VILI), colposcopy etc. and HPV DNA test by hybrid capture 2 (HC2), reverse line blot, real-time PCR etc., prevention and management. In order to evaluate the impact of awareness made available to participants, three times visits (1^st^ month, 3^rd^ month and 6^th^ month) was made to the same school. A semi-structured qualitative interview was conducted to collect the information regarding awareness, knowledge, disease exposome and vaccination programme etc. All the answers were recorded and reviewed. A good awareness response was observed among participants regarding the knowledge and awareness of cervical cancer and HPV. However, a negative response was documented with respect to implementation of vaccination programme in India due to adverse effects of vaccine related deaths recently. Consequently, a negative response which includes anxiety, physco-social distress and high cost of current vaccines was observed. In order to reduce the burden of cervical cancer and perhaps other disease prevalent in India, awareness through print and electronic – mass media is warranted.

## Discussion

As per our knowledge the present study is the first report to evaluate the knowledge of cervical cancer, HPV and HPV vaccine among the high-school and under graduate students in NCR (Noida) and Delhi region of India. It provides the useful information which may help in designing HPV vaccine programs and public health policy making in India. The school/college based studies of HPV awareness are very few and we observed an interesting correlation between increasing age and HPV awareness. Our result is in consistent with the other studies in India that showed low level of cervical cancer and HPV knowledge (15%) in graduate, postgraduate and even in medical students [24], [31]. But in contrast, another study from India showed that majority of the participants were well aware (89.6%) of several risk factors of cervical cancer development and its causal relation with HPV because of the study was conducted among medical students [32].

A study from Korea showed a mere 9.5% of female high school and university students had ever heard of HPV [33] But another study from Mexico among college students, aged 17–25 years, showed that most of the students have heard of HPV, although they had limited knowledge about the causal relation with virus and the preventive strategies [34]. Similarly, another study among college students aged 18–35 years in Ghana noted very low awareness (7.9%) regarding the link between HPV and cervical cancer [35]. Even a study from US showed that 21.5% of the college women have never heard of HPV [36]. Racial differences in HPV knowledge was also reported among US rural ethnic (29% in Blacks and 42% in Whites) populations [37]. Another study reported that 53.3% of the undergraduate female students had heard about cervical cancer and 37.8% knew about HPV in Durban, South Africa [38]. Similarly, another study from Taiwan among 953 undergraduate women aged 17–36 years in five universities in southern Taiwan reported that 70% of undergraduate women had limited knowledge of cervical cancer and 49% were having HPV awareness [39]. Therefore, it was reported that general public has low level of knowledge on HPV infection [20]. Similar to this study, another report from developing countries (India, Peru, Uganda, and Vietnam) showed low level of knowledge of HPV among children, parents, teachers, community leaders, and even health service providers of these four countries [40]. But in contrast, a study among the female educated youths in India, Nepal and Srilanka concluded that the awareness of cervical cancer was 66% in India, 58.8% in Nepal and 57.7% in Srilanka respectively [41]. Another study from Turkey among nursing students (age16–27 yrs) in tertiary hospital reported that they have theoretical knowledge but not aware that routine gynaecological examination and pap smears can be taken for prevention of cervical cancer [42]. There is a separate study in Polish population among female students (age 18–26 yrs) showed that general knowledge about cervical cancer and their causative agent of HPV is very high but they have poor knowledge about HPV vaccine and cytological screening [43]. But in contrast, a study from Greece among female University and technological institute students (age 18–26 years) reported that 59.1% students are aware of HPV vaccine and this high level of knowledge is positively associated with vaccine uptake [44].

In the present study, the male students were found to be less knowledgeable about HPV than the female students. This indicates that the female students were much aware about the cervical cancer. Similar to our findings, a study among Australian women and men between the ages 18 and 70 years showed that 62.8% of women and only 38.3% of men had heard of HPV [45]. In contrast, the studies from developing countries demonstrated that participants, aged 15–45 years, from both rural and urban settings have a low level of awareness about HPV [46]. Only one study from India among medical students showed that majority (89.6%) of the participates were aware of preventable nature of cervical cancer [32]. It was noticed from the other studies that gender also appears to have influenced knowledge and awareness, especially for HPV to some extent [47], [48]. Significant gender differences were observed, with females having better awareness and knowledge than males. Although the data are limited as not all studies reported results separately for males and females, these findings, could be reflective of the way awareness campaigns, for example on HPV, have been targeted more at females than at males.

The results of the present study also showed that Muslim students have very less knowledge as compared to Hindu students. It is important to note that presence of HPV and cervical cancer is relatively much lower in Muslim population. In this regards, the regional differences may be due to cultural, religious, social, ethnic variation [1]. Our results reflected that the HPV awareness is influenced by age, education, gender and community also.

The present study is also able to determine the parent’s opinion about HPV and HPV related information. Our previous study [26] demonstrated that parental literacy has a tremendous influence on the knowledge of cervical cancer (17%) and HPV infection (8%) for their school- aged children in India. In this study we found that knowledge about HPV infection causes genital cancer in male has little more knowledge than the female and in contrast the knowledge about cervical cancer is higher in females than in males. In another study from India reported that 41% participants were aware of a link between sexual activity and cervical cancer [24]. Similarly, a Korean survey [49] showed that 31.5% women aged more than 20 years were aware that sexually transmitted infections (STIs) can cause cervical cancer. But in contrast, few studies from Asian countries also reported of low knowledge levels of public on etiologic involvement of STIs and HPV in cervical cancer development [50], [51].

Study demonstrated that the area wise, urban dwellers had higher knowledge about “HPV infection causes genital cancer” than rural belt and similarly we found that knowledge about “cervical cancer” is higher in urban than rural. This study also showed that more attention is needed to educate the rural populations. In this regard, Li et al reported that (51.1%) urban women knew that HPV is related to cervical cancer in compare to their rural counterpart (41.6%). Even fewer (8.1%) knew that it is associated with genital warts with the similar rate of both geographies [52]. Poor knowledge and awareness of cervical cancer among women and other characteristics has been reported from many different geographic regions [35], [53], [54], [55]. This low level knowledge is consistent with the findings from another study of Chinese women in Hong Kong, which reported 10% of women were aware of HPV but had limited specific knowledge of HPV [51]. In some other developed countries with well integrated cervical cancer screening program with the Pap test, such as UK and US, the depth of knowledge about HPV were also reported to be very low [56], [57], [58]. In contrast, a relatively high rate of HPV awareness (51.2%) was reported from a study from Australia, which may be due to the increased media coverage, particularly in relation to the development of an HPV vaccination program [59]. As lack of knowledge is regarded as one of the major barriers that pose challenges to widespread implementation of HPV vaccine in developing countries [60], increased knowledge of education and health care providers; social workers may help to increase the general knowledge of HPV and HPV related diseases in India.

Our data represents that overall HPV vaccine awareness is very low both in female and male as well as dwellers of rural and urban origin. But in contrast, few studies reported that high vaccine acceptance among parents and adolescents in the general population [11], [57], [61], [62], [63], [64] found that 84% of participants would accept a free HPV vaccine, whereas 47% were unconcerned about future personal HPV infection risk. Males were less likely to accept a free HPV vaccine and to be concerned about future personal HPV infection risk. Among young women the acceptability of the vaccines is greater if they know that the latter give protection against genital warts [65]. But in most populations, knowledge of HPV and related vaccines is low but vaccine acceptance is high.

In our study majority of participants was unwilling to be vaccinated due to lack of knowledge of HPV vaccine and its safety which were the major reasons for them. This is consistent with studies from other areas and countries [14], [51], [66]. Our previous report [26] showed that some parents had diverse opinions that HPV vaccines would make sex safe, leading to freedom for promiscuity and risky sexual behaviour, which is not very common in this region of the globe due to sociocultural factors. They also thought that this would cause social stigmas and tarnish their family’s prestige, which has lead to widespread. Therefore, it is extremely important to raise general awareness about HPV, de-stigmatization of HPV infection and subsequently to gain acceptance for a mass vaccination program for pre-adolescent and adolescent girls in India.

In the present study parents decision between mother (15.8%) and father (8.3%) were very low about HPV vaccine as they are decision maker as vaccination takes place within the family unit, generally by one or both parents. We also observed that father’s roles in decision making are also important for finances or critical health events. Although several other studies have examined the preferences for the HPV vaccine among parents [19], [60], [66], [67], [68], [69] and among girls or young women [8], [47], [70], but none have compared parental preferences with adolescent preferences.

The strength of our study lies in the selection of study population of both rural and urban set up. But still there is limitation of our study. Findings of the present study could not be generalised to the large Indian population. Hence, an awareness regarding cervical cancer, its prevention and management is warranted.

In conclusion, in order to reduce the burden of cervical cancer and implementation of vaccination program, awareness is required which can be achieved by print and electronic media by raising slogans, conducing free camps in rural sectors for cancer screening and most importantly to sensitize Indian population for vaccine acceptance. Thus, there is a need with immediate effect to educate and aware the young population through print and/electronic media, NGOs, Hollywood, Bollywood stars and famous sports personalities about ill- myths associated with cervical cancer vaccination program in India.
